# Maternal and neonatal outcome of reverse breech extraction of an impacted fetal head during caesarean section in advanced stage of labour: a retrospective cohort study

**DOI:** 10.1186/s12884-019-2253-3

**Published:** 2019-03-27

**Authors:** Franziska Lenz, Nina Kimmich, Roland Zimmermann, Martina Kreft

**Affiliations:** 1 Department of Obstetrics and Gynaecology, Kantonsspital Baden AG, Baden, Switzerland; 2 Division of Obstetrics, University Hospital Zurich, Zurich, Switzerland

**Keywords:** Impacted fetal head, Reverse breech extraction, Caesarean section, Advanced stage of labour

## Abstract

**Background:**

Caesarean section with extraction of a deeply impacted fetal head is technically challenging and is associated with serious maternal and neonatal complications. The purpose of the study was to identify risks and evaluate selected outcome parameters associated with difficult fetal head extraction during caesarean section in advanced labour comparing two different extraction techniques (head pushing vs. reverse breech).

**Methods:**

This retrospective cohort study was conducted at the Division of Obstetrics in a tertiary care hospital in Zurich, Switzerland. 629 women at term with a singleton pregnancy in cephalic presentation during advanced intrapartum caesarean section from December 2012 until December 2016 were evaluated. Primary outcome was the incidence of uterine incision extensions. Secondary outcomes were other selected maternal and neonatal outcome parameters. Data analysis was performed using SPSS with Mann-Whitney U independent sampling test and two-tailed Fisher’s exact test (*p* < 0.01).

**Results:**

Difficult fetal head extractions are associated with significantly elevated maternal and neonatal risks. When performed by reverse breech technique, significant lower rates of extensions of the uterine incision, shorter operation times and less operative blood loss were identified compared to the head pushing method. No statistically significant differences for the neonatal outcomes were described so far. However, among the group of difficult fetal delivery with the head pushing method two neonates had perinatal skull fractures, with one of those resulting in neonatal death.

**Conclusions:**

The head pushing method is associated with higher maternal morbidity than the reverse breech method for extraction of a deeply engaged fetus during intrapartum caesarean section in advanced stage of labour.

## Background

Performing a caesarean section with extraction of a deeply impacted fetal head out of the maternal pelvis is technically challenging even for experienced obstetricians. The difficulty for the surgeon is to disengage the impacted head by hand due to a lack of space between the muscular and bony maternal pelvis and the deeply impacted fetal head [1]. This procedure is associated with elevated maternal risks including unintentional extensions of the uterine incision into the vascular broad ligament, prolonged operation times and postpartum haemorrhage [2–8]. Furthermore, serious neonatal complications, for instance skull injuries causing cerebral haemorrhage and newborn hypoxia that result in higher neonatal admission rates are described [1, 7, 9].

The constantly rising rates of intrapartum caesarean sections especially at full cervical dilation highlight the increasing importance of a skilful technique to deliver the fetus [10]. Thus, there are different methods of fetal delivery known in caesarean sections at advanced stage of labour. The conventional fetal extraction by lifting the head out of the maternal pelvis by the surgeon’s hand (head pushing) is often assisted by vaginal dislodge and exerts considerable force on the fetal head [5, 6, 11]. As an alternative to the head pushing technique, the pull method with fetal delivery via reverse breech was first described by Patwardhan 1957 [12] and is predominantly practiced in developing countries [5, 7–9, 13, 14]. The reverse breech technique was adopted according to the original description of Patwardhan [12] and established at our hospital for difficult fetal extractions during caesarean section in advanced labour since December 2014.

The objective of this study was to investigate selected maternal and neonatal outcome parameters of difficult deliveries with impacted fetal head during caesarean sections in obstructed labour with regard to different techniques of fetal extraction (head pushing vs. reverse breech). Therefore, we first identified obstetrical, maternal and neonatal risks that led to difficult fetal extractions. Second, we evaluated the outcomes associated with uncomplicated compared to difficult fetal extractions. Third, we examined the differences between the head pushing and the reverse breech method. As the primary outcome we analysed the incidence of uterine incision extensions, as secondary outcomes we selected maternal morbidities including operation time and operative blood loss as well as neonatal parameters.

## Methods

This retrospective study was conducted at the Division of Obstetrics in a tertiary care hospital in Zurich, Switzerland. A total number of 11,209 deliveries were recorded at our department between the observed time periods from December 2012 to December 2016. Among these deliveries 4928 caesarean sections were performed. We analysed all women at term (≥ 37 + 0 weeks of pregnancy) with a singleton pregnancy in cephalic presentation from our obstetric database, which required an intrapartum caesarean section at cervical dilation ≥7 cm between December 2012 and December 2016. The excluding criteria were multiple pregnancies, fetal anomalies, preterm delivery and fetal presentation other than cephalic. The final study population consisted of 629 women.

Obstetrical, maternal and fetal baseline criteria and outcomes were recorded and compared between groups of uncomplicated and difficult fetal extractions. The latter group was subdivided into deliveries performed by either the conventional head pushing method (*n* = 82) or the reverse breech technique (*n* = 55).

Cephalic malpresentation, as one of the baseline criteria, was defined as any fetal head presentation other than flexed occipito anterior. A larger fetal head circumference is determined to be a predictor for an unplanned caesarean section [15]. Lipschuetz et al. showed that a head circumference of > 35 cm increases the risk of a prolonged second stage of labour and is associated with a higher risk for an unplanned caesarean section [16]. Therefore, we selected a head circumference > 35 cm to evaluate a difference between the uncomplicated and difficult fetal extraction group.

The incision-delivery time is defined as the time interval between skin incision and delivery of the baby and the uterotomy-delivery time is understood to be the interval between uterus incision and the delivery of the baby. Both time intervals are routinely measured and documented when performing a caesarean section at our department.

A fetal extraction was defined to be difficult, when either T-incisions or transvaginal head pushing manoeuvres were performed, if the reverse breech extraction was necessary due to a failed fetal extraction caused by an impacted fetal head, or if declared as a difficult extraction in the surgical report (done by the surgeon just after having performed the caesarean section). Those information can be extracted out of our obstetric database, an in–house computerised patient data and clinical information system which contains the complete maternal, fetal and obstetrical data of every woman.

The conventional head pushing method during caesarean section was performed by lifting the fetal head out of the maternal pelvis by the surgeon’s hand. If the surgeon was not able to lift the head, vaginal dislodge by the help of an assistant’s hand or an inserted silicone cup (normally used for vacuum deliveries) was additionally performed, so the surgeon could finally deliver the fetal head through the uterine incision [6].

**Fig. 1:**
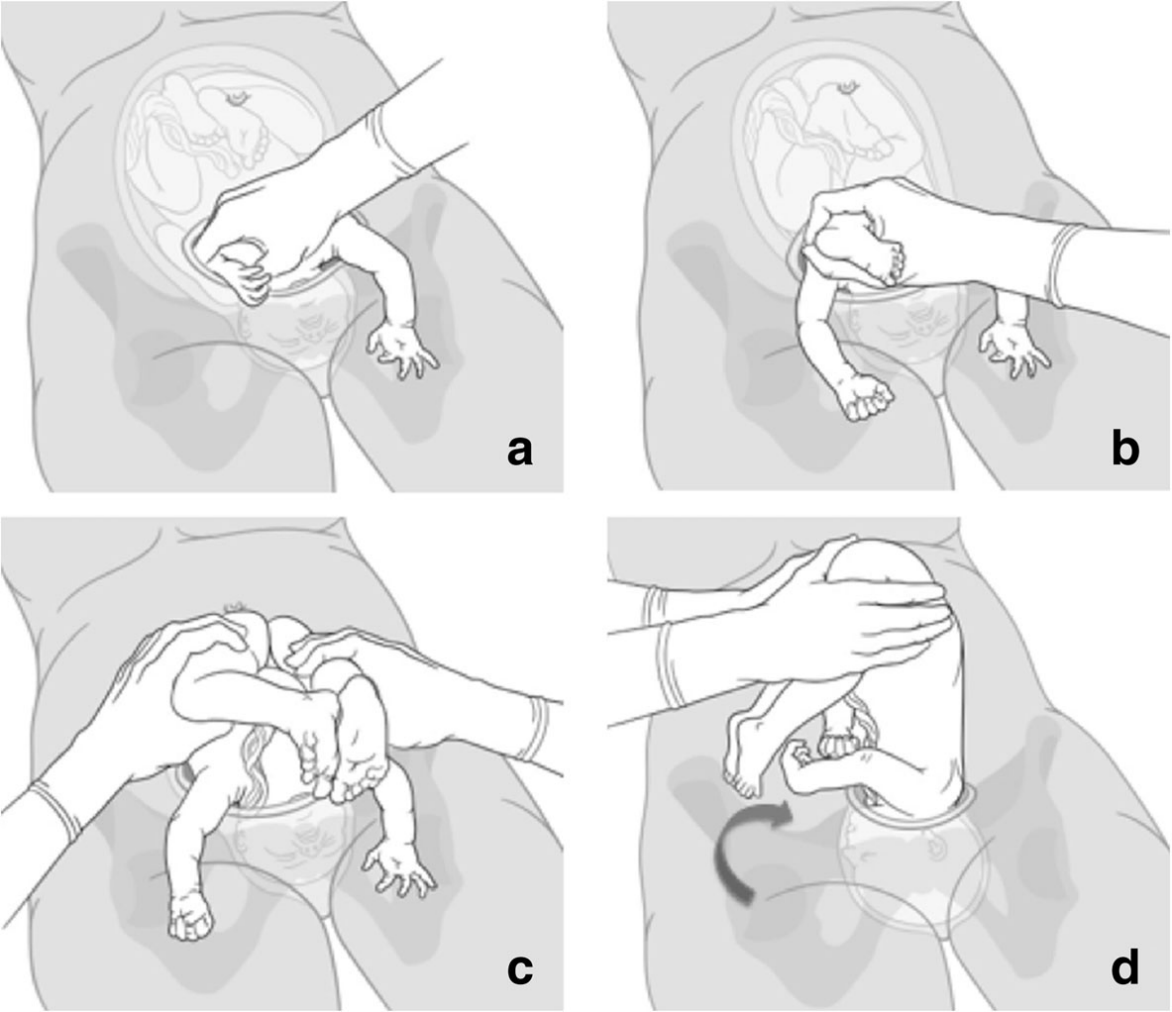
Reverse breech technique (occiput posterior position). **a** extract both fetal arms; **b** grasp the fetal foot with extraction of a leg; **c** extraction of the fetal body by pulling on both legs; **d** delivery of the fetal head by simultaneously screwing on the body and the shoulders

In comparison, the reverse breech extraction (pull method) is illustrated in Fig. 1, here with the fetus in occiput posterior position. After uterine incision first the fetal arms have to be extracted, than the surgeon grasps the feet and delivers both legs that are located in the fundal uterine region. After extraction of the fetal body by pulling symmetrically on both legs, sometimes supported by fundal pressure, the head can be easily disengaged from the maternal pelvis by an unscrewing manoeuvre [9, 17]. It should be taken into account that the fetal head when entering the maternal pelvis is positioned in a transverse diameter and rotates like a key in a keyhole from a transverse to an anterior posterior diameter. During caesarean section when the head is deeply impacted the process must be performed exactly backwards. Therefore, the head should be carefully unscrewed to the transverse diameter and possibly flexed so that the flexion point reaches the middle of the uterotomy. As the head is deeply impacted inside the maternal pelvis, it is initially not possible to grasp head and shoulders simultaneously. Therefore, when performing the reverse breech extraction the unscrewing is been done by first grasping the body and shoulders simultaneously and rotate the fetus very carefully. As soon as the fetal head can be reached, the shoulders and head are being grasped simultaneously so that the whole fetus can easily be delivered out of the uterotomy (or in assistance of Mauriceau-Smellie-Veit manoeuvre). This handling ensures a gentle delivery of the fetus and minimal twisting on the fetal spine.

**Fig. 2:**
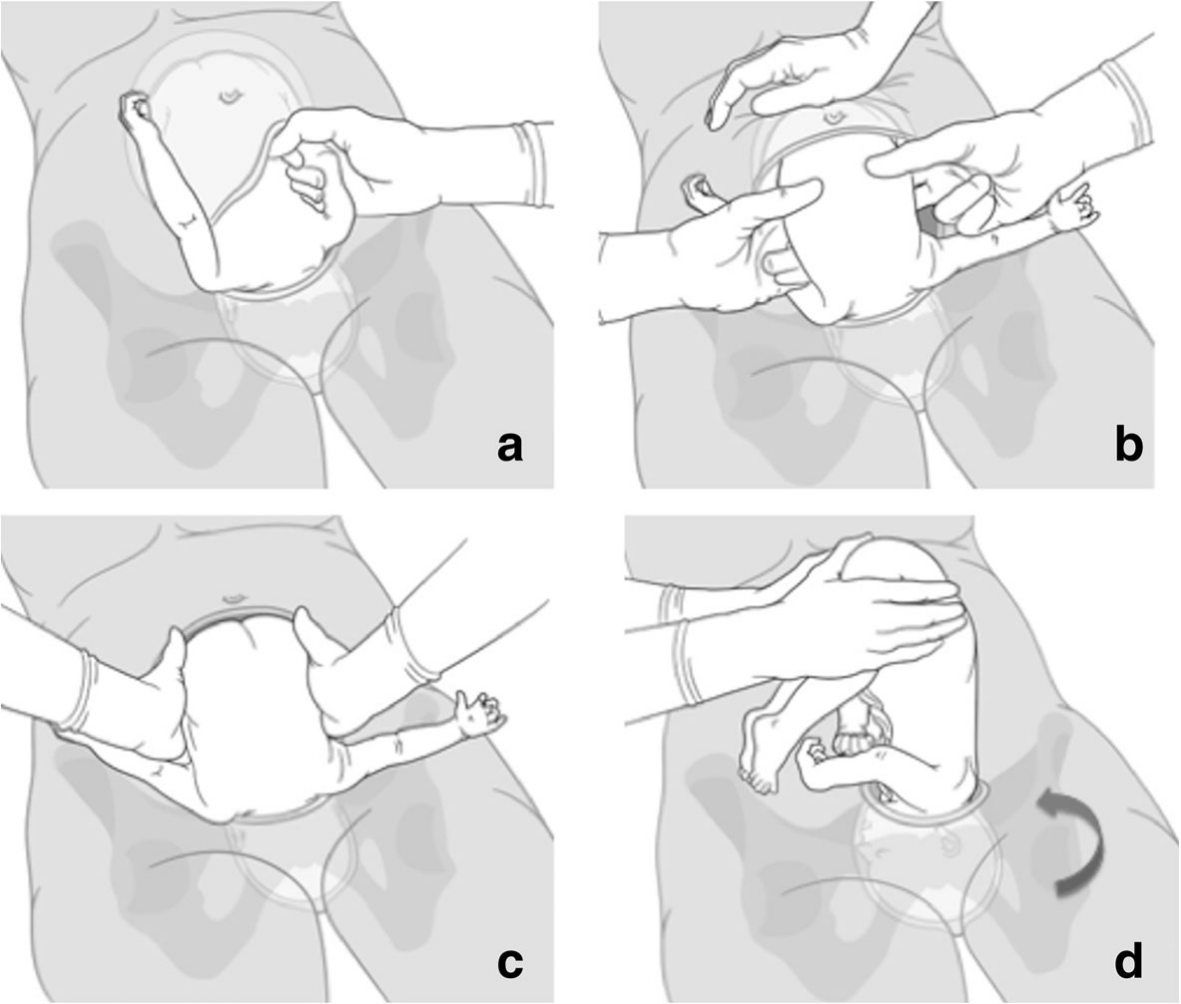
Reverse breech technique (occiput anterior position). **a** extract both fetal arms; **b** grasp the fetal trunk / hip with both hands; **c** extraction of the fetal body by pulling on the hips with pressure on the fundus; **d** delivery of the fetal head by simultaneously screwing on the body and the shoulders

Figure 2 shows the reverse breech technique in occiput anterior position. The surgeon grasps the babies back with both hands and pulls them gradually to reach the breech. Again, fundal pressure is sometimes helpful to extract the body. Thereafter, the identical unscrewing manoeuvre is applied to extract the head.

At our clinic unplanned caesarean sections are only performed by experienced residents under supervision of a senior consultant. Therefore, there is always an experienced team performing the caesarean section and in terms of difficult fetal extraction the consultant takes over to deliver the baby. Usually the experienced resident performs the surgery and tries to deliver the baby by lifting the head out of the maternal pelvis in case of vertex presentation. In case of difficult fetal extraction, the consultant takes over and first tries again to deliver the baby by lifting the head first. If the consultant is not able to get one hand under the fetal’s head, he then immediately grasps the fetal legs and performs a reverse breech delivery since the introduction of the new technique in December 2014.

The data was analysed using SPSS version 23 (IBM, Armonk, NY, USA). The Mann-Whitney U independent sampling test was used for continuous and the two-tailed Fisher’s exact test for categorical variables with *p* < 0.01 considered to indicate statistical significance.

## Results

When comparing uncomplicated and difficult fetal extractions a statistically significant difference in obstetrical and neonatal baseline criteria was seen in cervical dilation, rate of cephalic malpresentation and neonatal head circumference (Table 1).

**Table 1:** Baseline criteria comparing uncomplicated and difficult fetal extraction

	baseline criteria	group 1:	group 2:	*p*-value
	mean ± SD / n (%)	uncomplicated (*n* = 492)	difficult (*n* = 137)	
obstetrical	gestational age (in weeks)	39.8 ± 1.1	39.64 ± 1.1	0.03
	full cervical dilation (10 cm)	310 (63.0)	103 (75.2)	0.008*
	cephalic malpresentation	260 (52.9)	91 (66.4)	0.005*
maternal	age (in years)	31.7 ± 5.0	31.6 ± 5.1	0.934
	BMI before pregnancy (in kg/m^2^)	22.8 ± 4.0	23.5 ± 4.7	0.075
neonatal	weight (in g)	3555.9 ± 450.8	3453.2 ± 420.9	0.02
	head circumference > 35 cm	267 (54.3)	53 (38.7)	0.001*

*SD* standard deviation, *n (%)* number (per cent), *BMI* body mass index, *significant statistical difference between the groups (*p* < 0.01)

Selected outcome parameters of an uncomplicated and difficult fetal delivery were compared. The comparison showed statistically significant differences between both groups, which can be seen in (Table 2).

**Table 2:** Outcome parameters comparing uncomplicated and difficult fetal extraction

	outcome parameters mean ± SD / n (%)	group 1: uncomplicated (*n* = 492)	group 2: difficult (*n* = 137)	p**-**value
obstetrical	operation time (in min)	32.2 ± 11.1	42.2 ± 17.7	< 0.001*
	incision-delivery time (in min)	4.6 ± 2.0	6.9 ± 2.9	< 0.001*
	uterotomy-delivery time (in min)	1.3 ± 0.8	2.8 ± 1.5	< 0.001*
	additional instrumental delivery	3 (0.6)	7 (5.1%)	0.003*
maternal	blood loss (in ml)	556 ± 229.9	652.2 ± 322.9	< 0.001*
	delta haemoglobin pre−/postpartum (in g/l)	21.4 ± 11.6	25.8 ± 12.2	< 0.001*
	extensions of the uterine incision	68 (13.8)	34 (24.8)	0.004*
	T-incisions	0	7 (5.1)	< 0.001*
neonatal	umbilical arterial pH < 7.15	10 (2.0)	12 (8.8)	< 0.001*
	Apgar score at five minutes < 7	5 (1.0)	3 (2.2)	0.381
	admissions to the neonatal unit	1 (0.2)	3 (2.2)	0.01
	death	0	1 (0.7)	0.22

*SD* standard deviation, *n (%)* number (per cent), *BMI* body mass index, *significant statistical difference between the groups (*p* < 0.01)

Regarding the analysis of difficult fetal extractions by the two different methods of delivery (head pushing vs. reverse breech) no significant changes in baseline criteria could be identified (Table 3).

**Table 3:** Comparison of baseline criteria of difficult fetal delivery regarding the technique

	baseline criteria mean ± SD / n (%)	group 2a: head pushing (*n* = 82)	group 2b: reverse breech (*n* = 55)	p-value
obstetrical	gestational age (in weeks)	39.7 ± 1.0	39.5 ± 1.1	0.31
	full cervical dilation (10 cm)	58 (70.7)	45 (81.8)	0.162
	cephalic malpresentation	48 (58.5)	43 (78.1)	0.026
maternal	age (in years)	31.7 ± 5.0	31.6 ± 5.1	0.934
	BMI before pregnancy (in kg/m^2^)	23.9 ± 5.4	22.7 ± 2.9	0.116
neonatal	weight (in g)	3481.5 ± 382.5	3411 ± 473	0.359
	head circumference > 35 cm	33 (40.2)	20 (36.4)	0.722

*SD* standard deviation, *n (%)* number (per cent), *BMI* body mass index

Among the group of difficult extractions performed by reverse breech method we identified highly significant lower rates of extensions of the uterine incision, a shorter operation time and less blood loss compared to the conventional head pushing method during caesarean sections in advanced stage of labour (Table 4).

**Table 4:** Comparison of outcome parameters of difficult fetal delivery regarding the technique

	outcome parameters mean ± SD / n (%)	group 2a: head pushing (*n* = 82)	group 2b: reverse breech (*n* = 55)	p-value
obstetrical	operation time (in min)	44.8 ± 16.7	38.3 ± 18.4	0.006*
	incision-delivery time (in min)	7.0 ± 3.2	6.7 ± 2.5	0.979
	uterotomy-delivery time (in min)	2.7 ± 1.5	3.1 ± 1.5	0.085
	additional instrumental delivery	7 (8.5)	0	0.041
maternal	operative blood loss (in ml)	712.2 ± 375.0	562.7 ± 195.1	0.009*
	delta haemoglobin pre−/postpartum (in g/l)	26.8 ± 12.9	24.2 ± 10.9	0.257
	extensions of the uterine incision	29 (35.4)	5 (9.1)	< 0.001*
	T-incisions	5 (6.1)	2 (3.6)	0.702
neonatal	umbilical arterial pH < 7.15	8 (9.8)	4 (7.3)	0.768
	Apgar score at five minutes < 7	3 (3.7)	0	0.274
	transmissions to the neonatology unit	2 (2.4)	1 (1.8)	1.0
	death	1 (1.2)	0	1.0

*SD* standard deviation, *n (%)* number (per cent), *BMI* body mass index, *significant statistical difference between the groups (*p* < 0.01)

Between the groups there were no statistically significant differences with regard to fetal outcome, however all parameters tend to benefit from the reverse breech method. Concerning the neonatal outcome, there was only one single admission to the neonatal intensive care unit after reverse breech extraction due to acute respiratory distress syndrome. However, one fetal humours fracture was caused by a reverse breech extraction in occiput posterior position when the baby’s arm was hidden behind the back and it was technically impossible to extract it first. In this case no indication of primary admission to the neonatal intensive care unit was given. Among the group of difficult fetal delivery with the head pushing method two neonatal admissions were necessary due to perinatal skull fractures, with one of those resulting in neonatal death.

## Discussion

The deeply impacted fetal head is an obstetrical emergency situation, which requires a secure delivery technique to prevent undesirable maternal and neonatal consequences [1, 13].

Regarding a difficult fetal delivery, head pushing is the most commonly practised technique. However, reverse breech extraction has gradually been given higher priority, not only in developing countries with longer periods of second stage labour, but also in higher resource settings [1, 5, 6, 18, 19]. Rising rates of intrapartum caesarean sections and the presented significant differences of an uncomplicated compared to a difficult delivery highlight the importance of a safe intrapartum care for mother and child [6, 10]. On one hand, difficult fetal extractions are associated with an increased maternal risk of postpartum haemorrhage with elevated blood loss and higher delta haemoglobin pre- and postpartum [9, 13]. This increased risk results from a prolonged incision-delivery time, uterotomy-delivery time, total operation time and are caused by a higher rate of extensions of the uterine wound, T-incisions and additional instrumental support. On the other hand, difficult fetal extraction leads to severe neonatal consequences such as significantly higher rates of neonatal umbilical arterial pH < 7.15 and admissions to the neonatal care unit [1, 7, 9]. Therefore, it is important to incorporate alternative methods of fetal delivery into the daily obstetrical routine for a better outcome for mother and child.

With the introduction of the reverse breech method in caesarean sections for obstructed labour in 2014 we were able to observe less maternal complications with emphasis on a significantly lower rate of extensions of the uterine incision, which has been defined as the primary outcome (*p* < 0.001). Similar results regarding a higher rate of extensions of the uterine incision in caesarean sections performing fetal extraction via push technique were found in earlier publications [1, 5, 6, 8, 9, 14, 18, 20]. A shorter operation time and less blood loss compared to the head pushing method were also evaluated. The present findings correlate with the results of Sethuram et al. [6], Berhan & Berhan [7]_,_ and Veisi et al. [13] describing a significant rise of the duration of surgery and of uterine wound extensions, plus higher blood loss in the head pushing group among difficult fetal extractions by comparing the two mentioned delivery techniques.

No statistically significant findings in the present analysis were identified regarding neonatal outcome when comparing the two extraction methods. Compared to the conventional cephalic delivery, where two skull injuries were found that resulted in severe neonatal complications and even one in neonatal death, none were detected in the reverse breech group. Berhan & Berhan [7] report an increase of overall perinatal mortality in the head pushing group when compared to reverse breech. Concerning fetal birth trauma Fasubaa et al. [9], Veisi et al. [13], and Bastani et al. [20] did not describe any significant fetal differences between the two investigated extraction techniques. In contrast to the present data and former studies [8, 13, 14, 20] Fasubaa et al. [9] could also prove significant differences in fetal Apgar scores at five minutes and in the rates of neonatal death. However, similar to the results of former studies one extremity fracture caused by reverse breech extraction was described [6, 7, 13, 18]. This fact indicates the need for an even more skilful and gentle approach in the future.

The present baseline criteria of difficult fetal extractions in general showed that the risk of having a difficult fetal extraction during caesarean section rises with an increase of cervical dilation, especially when fully dilated. Furthermore, fetuses with larger head circumferences > 35 cm have a higher risk to not enter the deep pelvis in intrapartum caesarean sections most likely caused by cephalopelvic disproportion and therefore have a lower risk of being impacted in the maternal pelvis. On the other hand, fetuses with a head circumference below 35 cm or with cephalic malpresentation are more likely to have a difficult extraction caused by impaction in the maternal pelvis.

When comparing the different extraction methods (head pushing vs. reverse breech) significant differences in maternal outcome can be seen. In fact, prolonged labour increases the thinning of the lower uterine segment by an engaged fetal head and elevates the risk of damage to the uterine vessels and the lower urinary tract by cephalic delivery via head pushing method [3, 7, 13]. A highly significant reduced rate of extensions of the uterine incision may be explained by the more gentle delivery technique of reverse breech, which also results in a shorter operation time for repair, less blood loss due to less lacerations in the broad ligaments and less cervical lacerations, which has also been discussed in former studies [1, 3, 7, 13]. A shorter surgical duration also prevents a prolonged anaesthesia with potential side effects [12].

Regarding the neonatal outcome the present data showed less morbidity after reverse breech extraction compared to the head pushing method for obstructed labour. All outcomes show a tendency to a better effect in the reverse breech group. Further research with a higher number of cases is required to determine a significant difference definitely. Despite the lack of statistical significance the severity of neonatal morbidity shows clinical relevance. Originally, the reverse breech technique was also developed to improve the neonatal outcome, with the assumption that mainly tensile forces were acting and therefore the pressure on the child’s head could be reduced [9]. Thus, we suggest that the reverse breech technique should initially be considered in all intrapartum caesarean sections with a lack of space between the maternal pelvis and the impacted fetal head or when the anterior fetal arm has already dropped out after the uterine incision.

The 55 cases of reverse breech extraction had been analysed since the introduction of the new modified delivery method at our clinic. During this time the initially inexperienced obstetric staff had to pass a certain training period with appropriate written and practical instructions of reverse breech technique. At our institution we have different practical models of simulation training already. Desperate Debra® is one of those models to train difficult head extraction during caesarean section. A vaginal examination is possible to identify the fetal’s head position. The model allows rotation and flexion of the fetal head after insertion of a hand into the caesarean incision and between the fetal head and uterus. Because of missing shoulders and legs the reverse breech technique cannot be trained with this model. The development of a special training tool for reverse breech technique would improve the teaching and facilitate instructions to ensure a high level of confidence in a low-stress environment. Nevertheless, in order to introduce the reverse breech technique at our clinic we used other training tools for teaching such as lectures, hand-out material and own video tutorials made by the head of department while performing reverse breech technique by himself.

In the future, it will be necessary to further establish this pull technique in current daily practice and to intensify the training especially for inexperienced obstetrical staff to ensure safe intrapartum care and prove statistical relevance of the neonatal outcome. Training tools such as objective structured assessment tools, case-based discussions, video analysis and mini-clinical examinations for complex caesarean sections in obstructed labour are mandatory to improve the trainees` confidence and establish a clinical standard [6].

### Strengths

The present study was one of very few conducted in a tertiary hospital with validated standards compared to the majority of former studies taken from obstetric units in lower-resource settings or performed in developing countries. Evidence is limited, however with 629 included participants analysed during an observation period of 4 years, our representative trail has a large sample size of evaluated cases. A remarkable number of significances calculated from 55 cases of reverse breech extractions were found despite the fact that the staff still had to pass a learning period for this new modified technique.

### Limitations

The definition of a truly engaged fetal head resulting in a difficult extraction is subjective and always depends on the surgeon’s description. In this retrospective study two techniques of fetal extraction during caesarean section were analysed, however the risk of bias due to sometimes remarkable differences in experience, skills, and knowledge of the surgeons have to be taken into consideration. Additionally, when the reverse breech technique was first established at our clinic at the end of 2014, the obstetricians had to pass an individual learning curve to adapt to this new technique. Besides, no data of ultrasound during labour (for instance measurements of the angle of progression) were analysed to prove, whether an instrumental vaginal delivery would have been the better choice than a caesarean section in those cases where the cervix was fully dilated. Another limitation is a comparatively small number of rare outcomes such as fetal injuries, neonatal admissions to the intensive care unit or early neonatal death. To observe a statistically significant difference in neonatal outcome a much larger sample size is needed.

## Conclusions

The reverse breech method is associated with less maternal morbidity than the head pushing method for extraction of a deeply impacted fetal head during intrapartum caesarean delivery. The beneficial maternal-fetal results of performing the reverse breech procedure indicate that it is a reliable alternative to the standard head pushing method and should preferably be used in deeply impacted fetal head situations during caesarean section in advanced labour. Further randomised controlled trials are required to power especially the assessment of neonatal outcome and to confirm a suspected superiority of reverse breech method for both mother and child.

## Abbreviations

BMI: Body mass index
n: Number
SD: Standard deviation
